# Supplementary material for: The challenges of replication: A worked example of methods reproducibility using electronic health record data
Source: PLoS One. 2025 Jul 18;20(7):e0326335. doi: 10.1371/journal.pone.0326335 (PMC12273930; doi:10.1371/journal.pone.0326335)
Supplement: S1 File — (PDF) [file pone.0326335.s001.pdf]

CVD-COVID-UK / COVID-IMPACT Consortium Members (04/03/2025)

British Heart Foundation Data Science Centre
Led by Health Data Research UK

Institution	Member Name
Barts Health NHS Trust	Alastair Proudfoot
Barts Health NHS Trust	Andrew Constantine
Barts Health NHS Trust	Dan Jones
Barts Health NHS Trust	Krishnaraj Rathod
Barts Health NHS Trust	Nida Ahmed
Barts Health NHS Trust	Richard Fitzgerald
British Heart Foundation	Dan O'Connell
British Heart Foundation	Rony Arafin
British Heart Foundation	Sonya Babu-Narayan
British Heart Foundation	Zainab Karim
Cancer Research UK	Jon Shelton
Cancer Research UK	Martina Slapkova
Cancer Research UK	Rosie Hinchliffe
Cancer Research UK	Shane Johnson
Cardiff Metropolitan University	Renin Toms
Cardiff University	Julia Townson
European Bioinformatics Institute	Ewan Birney
European Bioinformatics Institute	Moritz Gerstung
European Bioinformatics Institute	Tomas Fitzgerald
Great Ormond Street Hospital	Katherine Brown
Guy's and St Thomas' NHS Foundation Trust	Benjamin Zuckerman
Guy's and St Thomas' NHS Foundation Trust	Ernest Wong
Guy's and St Thomas' NHS Foundation Trust	Lily Benton
Guy's and St Thomas' NHS Foundation Trust	Tasanee Braithwaite
Health Data Research UK	Alexis Webb
Health Data Research UK/BHF Data Science Centre	Anna Stevenson
Health Data Research UK/BHF Data Science Centre	Cathie Sudlow
Health Data Research UK/BHF Data Science Centre	Fionna Chalmers
Health Data Research UK/BHF Data Science Centre	Jadene Lewis
Health Data Research UK/BHF Data Science Centre	James Farrell
Health Data Research UK/BHF Data Science Centre	John Nolan

Institution	Member Name
Health Data Research UK/BHF Data Science Centre	Lars Murdock
Health Data Research UK/BHF Data Science Centre	Laura Sherlock
Health Data Research UK/BHF Data Science Centre	Lynn Morrice
Health Data Research UK/BHF Data Science Centre	Mehrdad Mizani
Health Data Research UK/BHF Data Science Centre	Melissa Webb
Health Data Research UK/BHF Data Science Centre	Ross Forsyth
Health Data Research UK/BHF Data Science Centre	Rouven Priedon
Health Data Research UK/BHF Data Science Centre	Samaira Khan
Health Data Research UK/BHF Data Science Centre	Sandy Wibrew
Health Data Research UK/BHF Data Science Centre	Steffen Petersen
Health Data Research UK/BHF Data Science Centre	Thomas Bolton
Health Data Research UK/BHF Data Science Centre	Zach Welshman
Healthcare Quality Improvement Partnership	Caroline Rogers
Hull York Medical School	Donna Wakefield
Imperial College London	Alun Davies
Imperial College London	Arunashis Sau
Imperial College London	Costas Kallis
Imperial College London	Fu Siong Ng
Imperial College London	Hannah Whittaker
Imperial College London	Ioanna Tzoulaki
Imperial College London	Jennifer Quint
Imperial College London	Juliette Unwin
Imperial College London	Libor Pastika
Imperial College London	Petter Brodin
Imperial College London	Philip Stone
Imperial College London	Safa Salim
Imperial College London	Sarah Cook
Imperial College London	Sarah Onida
Imperial College London (LCP)	Alistair Marsland

Institution	Member Name
Imperial College London (LCP)	Andrew Thompson
Imperial College London (LCP)	Sara Holloway
Imperial College London (LCP)	Thomas Porter
INSIGHT	Alastair Denniston
Keele University	Mamas Mamas
King's College London	Abdel Douiri
King's College London	Adejoke Oluyase
King's College London	Ajay Shah
King's College London	Alexandru Dregan
King's College London	Amy Ronaldson
King's College London	Anna Bone
King's College London	Antonio Cannata
King's College London	Ben Bray
King's College London	Charles Wolfe
King's College London	Daniel Bromage
King's College London	Dominic Oliver
King's College London	Elena Nikiphorou
King's College London	Emeka Chukwusa
King's College London	Gareth Williams
King's College London	Gayan Perera
King's College London	Harry Watson
King's College London	Iain Marshall
King's College London	Irene Higginson
King's College London	Javiera Leniz Martelli
King's College London	Jayati Das-Munshi
King's College London	Joanna Davies
King's College London	Johnny Downs
King's College London	Katherine Sleeman
King's College London	Laia Becares
King's College London	Linglong Qian
King's College London	Matthew O'Connell
King's College London	Mevhibe Hocaoglu
King's College London	Natasha Chilman

Institutions party to the Data Sharing Agreement with NHS England for access to the data in the Secure Data Environment service for England for CVD-COVID-UK/COVID-IMPACT are: Imperial College London, King's College London, London School of Hygiene & Tropical Medicine, Swansea University, University College London, University of Bristol, University of Cambridge, University of Dundee, University of Glasgow, University of Leicester, University of Liverpool, University of Manchester, University of Nottingham, University of Oxford, University of Sheffield, University of Southampton.

CVD-COVID-UK / COVID-IMPACT Consortium Members (04/03/2025)

British Heart Foundation Data Science Centre
Led by Health Data Research UK

Institution	Member Name
**King's College London**	Rachel Cripps
**King's College London**	Richard Killick
**King's College London**	Theresa McDonagh
**King's College London**	Vasa Curcin
**King's College London**	Zeljko Kraljevic
**Leeds Teaching Hospitals NHS Trust**	Carin van Doorn
**London School of Economics & Political Science**	Rocco Friebel
**London School of Hygiene & Tropical Medicine**	Antonio Gasparrini
**London School of Hygiene & Tropical Medicine**	Arturo de la Cruz
**London School of Hygiene & Tropical Medicine**	Dorothea Nitsch
**London School of Hygiene & Tropical Medicine**	Julian Matthewman
**London School of Hygiene & Tropical Medicine**	Neil Pearce
**London School of Hygiene & Tropical Medicine**	Patrick Bidulka
**London School of Hygiene & Tropical Medicine**	Qiuju Li
**London School of Hygiene & Tropical Medicine**	Sinéad Langan
**London School of Hygiene & Tropical Medicine**	Thiago Silva
**London School of Hygiene & Tropical Medicine**	Zhengnan Zhu
**Manchester University NHS Foundation Trust**	Martin Rutter
**Newcastle-upon-Tyne Hospitals NHS Foundation Trust**	Alex Grundmann
**NHS England**	Adam Hollings
**NHS England**	Angeliki Antonarou
**NHS England**	Daniel Schofield
**NHS England**	Deborah Lowe
**NHS England**	Elizabeth Kelly
**NHS England**	Richardson
**NHS England**	Humaira Hussein
**NHS England**	Jake Kasan
**NHS England**	Nickie Wareing
**NHS England**	Russell Healey
**NHS England**	Shoaib Ali Ajaib
**NHS Lanarkshire**	Mark Barber
**NHS Scotland**	Carole Morris
**NICE**	Felix Greaves
**NICE**	Jennifer Beveridge
**NICE**	Seamus Kent
**NICE**	Thomas Lawrence
**NICE**	Vandana Ayyar-Gupta
**Office for National Statistics**	Myer Glickman
**Office for National Statistics**	Vahé Nafilyan
**Queen Mary University of London**	Deepti Gurdasani
**Queen's University Belfast**	Frank Kee
**Royal Brompton and Harefield Hospitals**	Paz Tayal
**Royal College of Surgeons of England**	David Cromwell
**Royal Free London NHS Foundation Trust**	Amar Shah
**Royal Free London NHS Foundation Trust**	Swapna Mandal
**Royal Papworth Hospital NHS Foundation Trust**	Florian Falter
**Royal Papworth Hospital NHS Foundation Trust**	Joseph Newman
**Royal United Hospitals Bath NHS Foundation Trust**	Jennifer Rossdale
**St George's University of London**	Baboucarr Njie
**St George's University of London**	Elijah Behr
**St George's University of London**	Nuria Sanchez
**St George's University of London**	Xinkai Wang
**Swansea Bay University Health Board**	Daniel Harris
**Swansea University**	Amanda Marchant
**Swansea University**	Ashley Akbari
**Swansea University**	Daniel King
**Swansea University**	David Powell
**Swansea University**	Elizabeth A Ellins
**Swansea University**	Fatemeh Torabi
**Swansea University**	Gareth Davies
**Swansea University**	Hoda Abbasizanjani
**Swansea University**	Huw Strafford
**Swansea University**	Jane Lyons
**Swansea University**	Julian Halcox
**Swansea University**	Laura North
**Swansea University**	Marcos del Pozo Banos
**Swansea University**	Owen Pickrell
**Swansea University**	Ronan Lyons
**Swansea University - DATAMIND**	Ann John
**University College**	Robert Aldridge
**University College London**	Abraham Olvera-Barrios
**University College London**	Adnan Tufail
**University College London**	Alasdair Warwick
**University College London**	Alex Handy
**University College London**	Alexei Yavlinsky
**University College London**	Alvina Lai
**University College London**	Ami Banerjee
**University College London**	Ana Torralbo
**University College London**	Ana-Catarina Pinho-Gomes
**University College London**	Andrej Ivanovic
**University College London**	Andrew Lambarth
**University College London**	Anthony Khawaja
**University College London**	Ashkan Dashtban
**University College London**	Ashley Dickson
**University College London**	Becky White
**University College London**	Christina Pagel
**University College London**	Christopher Tomlinson
**University College London**	Chu Siyu
**University College London**	David Selby
**University College London**	Eloise Withnell
**University College London**	Emma Whitfield
**University College London**	Eva Keller
**University College London**	Evaleen Malgapo
**University College London**	Ferran Espuny-Pujol
**University College London**	Flavien Hardy
**University College London**	Floriaan Schmidt
**University College London**	Freya Allery
**University College London**	Harry Hemingway

Institutions party to the Data Sharing Agreement with NHS England for access to the data in the Secure Data Environment service for England for CVD-COVID-UK/COVID-IMPACT are: Imperial College London, King's College London, London School of Hygiene & Tropical Medicine, Swansea University, University College London, University of Bristol, University of Cambridge, University of Dundee, University of Glasgow, University of Leicester, University of Liverpool, University of Manchester, University of Nottingham, University of Oxford, University of Sheffield, University of Southampton.

CVD-COVID-UK / COVID-IMPACT Consortium Members (04/03/2025)

British Heart Foundation Data Science Centre
Led by Health Data Research UK

Institution	Member Name
**University College London**	Honghan Wu
**University College London**	Jinge Wu
**University College London**	Johan Thygesen
**University College London**	Johannes Heyl
**University College London**	Julia Ive
**University College London**	Kate Cheema
**University College London**	Katie Harron
**University College London**	Ken Li
**University College London**	Kerrie Stevenson
**University College London**	Laura Pasea
**University College London**	Louise Choo
**University College London**	Luca Grieco
**University College London**	Manuel Gomes
**University College London**	Matt Sydes
**University College London**	Mehrdad Mizani
**University College London**	Michalis Katsoulis
**University College London**	Mohamed Mohamed
**University College London**	Naomi Launders
**University College London**	Nushrat Khan
**University College London**	Paula Lorgelly
**University College London**	Pedro Machado
**University College London**	Pia Hardelid
**University College London**	Qi Huang
**University College London**	Riyaz Patel
**University College London**	Roy Schwartz
**University College London**	Rui Providencia
**University College London**	Ruth Gilbert
**University College London**	Samuel Kim
**University College London**	Simon Ellershaw
**University College London**	Sonya Crowe
**University College London**	Spiros Denaxas
**University College London**	Tuankasfee Hama
**University College London**	Waty Lilaonitkul

Institution	Member Name
**University College London**	Yi Mu
**University College London**	Yohhei Hamada
**University College London**	Yoryos Lyratzopoulos
**University College London / DATAMIND**	David Osborn
**University Hospital of North Midlands**	Arun Pherwani
**University of Aberdeen**	Mary Joan Macleod
**University of Birmingham**	Sarah Wang
**University of Birmingham and Queen Elizabeth Hospital Birmingham**	Mark Thomas
**University of Bristol**	Arun Karthikeyan Suseeladevi
**University of Bristol**	Ben Gibbison
**University of Bristol**	Dann Mitchell
**University of Bristol**	Deborah Lawler
**University of Bristol**	Eleanor Walsh
**University of Bristol**	Elsie Horne
**University of Bristol**	Ewan Walker
**University of Bristol**	Gianni Angelini
**University of Bristol**	Jeremy Chan
**University of Bristol**	John Macleod
**University of Bristol**	Jonathan Sterne
**University of Bristol**	Katharine Looker
**University of Bristol**	Kurt Taylor
**University of Bristol**	Livia Pierotti
**University of Bristol**	Luisa Zuccolo
**University of Bristol**	Martha Elwenspoek
**University of Bristol**	Marwa Al Arab
**University of Bristol**	Massimo Caputo
**University of Bristol**	Mira Hidajat
**University of Bristol**	Neil Davies
**University of Bristol**	Paul Madley-Dowd
**University of Bristol**	Rachel Denholm
**University of Bristol**	Rochelle Knight
**University of Bristol**	Rupert Payne

Institution	Member Name
**University of Bristol**	Shubhra Sinha
**University of Bristol**	Tim Dong
**University of Bristol**	Tom Palmer
**University of Bristol**	Venexia Walker
**University of Bristol**	Yueying Li
**University of Bristol**	Yvonne Nartey
**University of Cambridge**	Alexia Sampri
**University of Cambridge**	Angela Wood
**University of Cambridge**	Carmen Petitjean
**University of Cambridge**	Chimweta Chilala
**University of Cambridge**	Chriselda Oliver
**University of Cambridge**	David Brind
**University of Cambridge**	Elena Raffetti
**University of Cambridge**	Elias Allara
**University of Cambridge**	Emanuele Di Angelantonio
**University of Cambridge**	Eoin McKinney
**University of Cambridge**	Eric Harshfield
**University of Cambridge**	Fabian Falck
**University of Cambridge**	Genevieve Cezard
**University of Cambridge**	Hannah Harrison
**University of Cambridge**	Haoting Zhang
**University of Cambridge**	Holly Pavey
**University of Cambridge**	Isabel Walter
**University of Cambridge**	Jessica Barrett
**University of Cambridge**	John Danesh
**University of Cambridge**	John Ford
**University of Cambridge**	Katie Saunders
**University of Cambridge**	Lisa Pennells
**University of Cambridge**	Lois Kim
**University of Cambridge**	Matilda Pitt
**University of Cambridge**	Mayank Dalakoti
**University of Cambridge**	Megan Ritson
**University of Cambridge**	Mike Inouye

Institutions party to the Data Sharing Agreement with NHS England for access to the data in the Secure Data Environment service for England for CVD-COVID-UK/COVID-IMPACT are: Imperial College London, King's College London, London School of Hygiene & Tropical Medicine, Swansea University, University College London, University of Bristol, University of Cambridge, University of Dundee, University of Glasgow, University of Leicester, University of Liverpool, University of Manchester, University of Nottingham, University of Oxford, University of Sheffield, University of Southampton.

CVD-COVID-UK / COVID-IMPACT Consortium Members (04/03/2025)

British Heart Foundation Data Science Centre
Led by Health Data Research UK

Institution	Member Name
University of Cambridge	Millie Zhou
University of Cambridge	Pearl Liu
University of Cambridge	Robert Fletcher
University of Cambridge	Rutendo Mapeta
University of Cambridge	Samantha Ip
University of Cambridge	Spencer Keene
University of Cambridge	Stelios Boulitsakis Logothetis
University of Cambridge	Stephen Kaptoge
University of Cambridge	Tianxiao Wang
University of Cambridge	Tom Pape
University of Cambridge	Wen Shi
University of Cambridge	Xilin Jiang
University of Cambridge	Xiyun Jiang
University of Cambridge	Yanfan Li
University of Dundee	Daniel Morales
University of Dundee	Huan Wang
University of Dundee	Ify Mordi
University of Dundee	Samira Bell
University of Edinburgh	Alan Carson
University of Edinburgh	Alice Hosking
University of Edinburgh	Annemarie Docherty
University of Edinburgh	Athina Spiliopoulou
University of Edinburgh	Baljean Dhillon
University of Edinburgh	Carlos Sanchez Soriano
University of Edinburgh	Caroline Jackson
University of Edinburgh	Christian Schnier
University of Edinburgh	Claire Tochel
University of Edinburgh	Gwenetta Curry
University of Edinburgh	Helen Colhoun
University of Edinburgh	Joe Mellor
University of Edinburgh	Kelly Fleetwood
University of Edinburgh	Laura Sherlock
University of Edinburgh	Luke Blackbourn
University of Edinburgh	Michelle Williams
University of Edinburgh	Miguel Bernabeu Llinares
University of Edinburgh	Niamh McLennan
University of Edinburgh	Rebecca Reynolds
University of Edinburgh	Richard Chin
University of Edinburgh	Steven Kerr
University of Edinburgh	Tim Wilkinson
University of Edinburgh	Verónica Cabreira
University of Edinburgh	William Berthon
University of Edinburgh	William Whiteley
University of Exeter	John Dennis
University of Exeter	Kerry Pearn
University of Exeter	Michael Allen
University of Glasgow	Angela Henderson
University of Glasgow	Clea du Toit
University of Glasgow	Colin Berry
University of Glasgow	Craig Melville
University of Glasgow	Deborah Kinnear
University of Glasgow	Dennis Tran
University of Glasgow	Filip Sosenko
University of Glasgow	Frederick Ho
University of Glasgow	Jill Pell
University of Glasgow	Jocelyn Friday
University of Glasgow	John Cleland
University of Glasgow	Naveed Sattar
University of Glasgow	Ninian Lang
University of Glasgow	Salil Deo
University of Glasgow	Sandosh Padmanabhan
University of Glasgow	Terry Quinn
University of Leeds	Jianhua Wu
University of Leeds	Marlous Hall
University of Leeds	Ramesh Nadarajah
University of Leicester	Anna Hansell
University of Leicester	Anvesha Singh
University of Leicester	Cameron Razieh
University of Leicester	Claire Lawson
University of Leicester	Clare Gillies
University of Leicester	Francesco Zaccardi
University of Leicester	Iain Squire
University of Leicester	Kamlesh Khunti
University of Leicester	Matthew Bown
University of Leicester	Muhammad Rashid
University of Leicester	Sharmin Shabnam
University of Leicester	Shirley Sze
University of Leicester	Tom Yates
University of Leicester	Yogini Chudasama
University of Liverpool	Andrew Mason
University of Liverpool	Benedict Michael
University of Liverpool	Caroline Dale
University of Liverpool	David Hughes
University of Liverpool	Francesca Zaccagnino
University of Liverpool	Ike Chukwudi
University of Liverpool	Maria Sudell
University of Liverpool	Mark Green
University of Liverpool	Munir Pirmohamed
University of Liverpool	Pardis Biglarbeigi
University of Liverpool	Reecha Sofat
University of Liverpool	Rohan Takhar
University of Liverpool	Ruwanthi Kolamunnage-Dona
University of Liverpool	Stephen McKeever
University of Manchester	Bernard Keavney
University of Manchester	Catriona Harrison
University of Manchester	Craig Smith
University of Manchester	David Jenkins
University of Manchester	Eva Henning
University of Manchester	Evan Kontopantelis

Institutions party to the Data Sharing Agreement with NHS England for access to the data in the Secure Data Environment service for England for CVD-COVID-UK/COVID-IMPACT are: Imperial College London, King's College London, London School of Hygiene & Tropical Medicine, Swansea University, University College London, University of Bristol, University of Cambridge, University of Dundee, University of Glasgow, University of Leicester, University of Liverpool, University of Manchester, University of Nottingham, University of Oxford, University of Sheffield, University of Southampton.

CVD-COVID-UK / COVID-IMPACT Consortium Members (04/03/2025)

British Heart Foundation Data Science Centre
Led by Health Data Research UK

Institution	Member Name
University of Manchester	George Tilston
University of Manchester	Glen Martin
University of Manchester	Hector Chinoy
University of Manchester	Joseph Firth
University of Manchester	Lana Bojanić
University of Manchester	Matthew Sperrin
University of Manchester	Max Lyon
University of Manchester	Maya Buch
University of Manchester	Richard Williams
University of Manchester	Ruth Norris
University of Manchester	Ruth Watkinson
University of Manchester	Sarah Steeg
University of Manchester	Simon Frain
University of Manchester	Simon Williams
University of Manchester	Steven Zhao
University of Manchester	Zenas Yiu
University of Newcastle	Camille Carroll
University of Newcastle	Charlotte Parbery-Clark
University of Newcastle	Dexter Canoy
University of Newcastle	Precious Onyeachu
University of Nottingham	Fiona Pearce
University of Nottingham	Laila Tata
University of Nottingham	Ralph Akyea
University of Nottingham	Stephanie Lax
University of Oxford	Aashna Uppal
University of Oxford	Akshay Shah
University of Oxford	Antonella Delmestri
University of Oxford	Antony Palmer
University of Oxford	Ben Goldacre
University of Oxford	Ben Lacey
University of Oxford	Dani Prieto-Alhambra
University of Oxford	Eva Morris
University of Oxford	George Nicholson
University of Oxford	Hayley Evans
University of Oxford	James Sheppard
University of Oxford	Joseph Kamtchum Tatuene
University of Oxford	Julia Hippisley-Cox
University of Oxford	Kazem Rahimi
University of Oxford	Linxin Li
University of Oxford	Lucy Wright
University of Oxford	Marta Pineda Moncusi
University of Oxford	Mohammad Mamouei
University of Oxford	Nick Hall
University of Oxford	Parag Gajendragadkar
University of Oxford	Paula Dhiman
University of Oxford	Qingze Gu
University of Oxford	Raph Goldacre
University of Oxford	Salma Chaudhry
University of Oxford	Sara Khalid
University of Oxford	Seb Bacon
University of Oxford	Seyed Alireza Hasheminasab
University of Oxford	Shishir Rao
University of Oxford	Xiaomin Zhong
University of Oxford	Zeinab Bidel Taleshmekaeil
University of Oxford/KU Leuven	Nathalie Conrad
University of Plymouth	Marie-Louise Zeissler
University of Sheffield	Jen-Yu Amy Chang
University of Sheffield	Norman Briffa
University of Sheffield	Peter Bath
University of Sheffield	Simone Croft
University of Sheffield	Suzanne Mason
University of Sheffield	Tim Chico
University of Southampton	Nazrul Islam
University of Strathclyde	Amanj Kurdi
University of Strathclyde	Kim Kavanagh
University of Strathclyde	Marion Bennie
University of Strathlcyde	Tanja Mueller
University of Warwick	Harry Wilde
University of Warwick	Majel McGranahan
University of York	Christina van der Feltz-Cornelis
University of York	Han-I Wang
University of York	Lorna Fraser
Wellcome Sanger Institute	Tapiwa Tungamirai

Institutions party to the Data Sharing Agreement with NHS England for access to the data in the Secure Data Environment service for England for CVD-COVID-UK/COVID-IMPACT are: Imperial College London, King's College London, London School of Hygiene & Tropical Medicine, Swansea University, University College London, University of Bristol, University of Cambridge, University of Dundee, University of Glasgow, University of Leicester, University of Liverpool, University of Manchester, University of Nottingham, University of Oxford, University of Sheffield, University of Southampton.
