# Supplementary material for: The challenges of replication: A worked example of methods reproducibility using electronic health record data
Source: PLoS One. 2025 Jul 18;20(7):e0326335. doi: 10.1371/journal.pone.0326335 (PMC12273930; doi:10.1371/journal.pone.0326335)
Supplement: Attachment — Submitted filename: Response to reviewers.odt [file pone.0326335.s003.odt]

Additional Editor Comments:
 
The paper is of significant value and can be considered for publication pending minor revisions in accordance with the reviewer's suggestions.
Thank you for the positive review. The time taken is much appreciated. We will endeavour to respond appropriately to the comments below.
Please ensure that your manuscript meets PLOS ONE's style requirements, including those for file naming. 
We have made changes around headings and sub-headings to bring the manuscript in line with PLOS ONE’s style requirements.
One of the noted authors is a group or consortium [CVD-COVIDUK/COVID-IMPACT]. In addition to naming the author group, please list the individual authors and affiliations within this group in the acknowledgments section of your manuscript. Please also indicate clearly a lead author for this group along with a contact email address.
The consortium has about 500 members so I’m not sure if it’s appropriate to list each member. Any members who contributed sufficiently to warrant authorship are already in the author list. The style guidance says a link to an internet resource is sufficient, so we have linked to the page on the BHF website that lists all current consortium members.
Any changes to the reference list should be mentioned in the rebuttal letter that accompanies your revised manuscript.
We have changed one of the citations. Previously we had cited an article on MedArxiv. This has now been published and so we have updated the link to point to the published article instead.
Thank you for stating the following in the Acknowledgments Section of your manuscript:
[This work is carried out with the support of the BHF Data Science Centre led by HDR UK (BHF Grant no. SP/19/3/34678).]
We note that you have provided funding information that is not currently declared in your Funding Statement. However, funding information should not appear in the Acknowledgments section or other areas of your manuscript. We will only publish funding information present in the Funding Statement section of the online submission form.
Please remove any funding-related text from the manuscript and let us know how you would like to update your Funding Statement. Currently, your Funding Statement reads as follows:
[The author(s) received no specific funding for this work.]
Please include your amended statements within your cover letter; we will change the online submission form on your behalf.
We have removed the funding statement from the manuscript, and the line in the acknowledgments that mentioned funding. The full funding statement should be:
The British Heart Foundation Data Science Centre (grant No SP/19/3/34678, awarded to Health Data Research (HDR) UK) funded co-development (with NHS England) of the Secure Data Environment service for England, provision of linked datasets, data access, user software licences, computational usage, and data management and wrangling support, with additional contributions from the HDR UK Data and Connectivity component of the UK Government Chief Scientific Adviser’s National Core Studies programme to coordinate national COVID-19 priority research. Consortium partner organisations funded the time of contributing data analysts, biostatisticians, epidemiologists, and clinicians. 
The associated costs of accessing data in NHS England’s secure data environment service for England, for analysts working on this study, were funded by the Data and Connectivity National Core Study, led by Health Data Research UK in partnership with the Office for National Statistics, which is funded by UK Research and Innovation (grant ref: MC_PC_20058). 
This research was co-funded by the NIHR Manchester Biomedical Research Centre (NIHR203308) and the NIHR Applied Research Collaboration Greater Manchester (NIHR200174). AMW is supported by the BHF Data Science Centre (HDRUK2023.0239) and as an NIHR Research Professor (NIHR303137). This work was supported by core funding from the: British Heart Foundation (RG/18/13/33946), NIHR Cambridge Biomedical Research Centre (BRC-1215-20014; NIHR203312), Cambridge BHF Centre of Research Excellence (RE/18/1/34212), BHF Chair Award (CH/12/2/29428) and by Health Data Research UK, which is funded by the UK Medical Research Council, Engineering and Physical Sciences Research Council, Economic and Social Research Council, Department of Health and Social Care (England), Chief Scientist Office of the Scottish Government Health and Social Care Directorates, Health and Social Care Research and Development Division (Welsh Government), Public Health Agency (Northern Ireland), British Heart Foundation and Wellcome. 
The views expressed are those of the author(s) and not necessarily those of the NIHR or the Department of Health and Social Care. 
Reviewers' comments:
 
Reviewer #1: I congratulate the authors on their interesting paper on a working example of a reproducibility study with electronic health record (EHR) data.
I found the paper very well written and IMO the paper discusses very important challenges and possible solutions, especially for the problem of data reproducibility. In the worked example, the challenge of reproducing the results is not in the data analysis or statistical analysis part, as all the scripts relevant for these analyses are available and applicable, provided that the data tables are set up exactly as in the study for which a replication study is planned. The challenge is to obtain the data in the same structure from different databases. The conclusions are summarised in 12 recommendations for so-called Trusted Research Environments (TREs) and Secure Data Environments (SDEs) to manage such replication studies.
Thank you for taking the time to review our paper, and for your very positive comments. We hope the changes made in response to your comments further strengthen the paper.
 
A drawback of the paper is certainly that the example study presented is very much tailored to how the UK system (NHS) manages access to EHR data. However, I think that the recommendations can be applied in a similar way to health systems in many countries, i.e. I suspect that there is also value in reading about the challenges for research groups not based in the UK.
 
I suggest that the authors write a paragraph on how they think their 12 recommendations can be generalised to other health systems (although they have described this problem in a limitation paragraph). This would add value to the paper.
An earlier version of the paper did indeed make this generalisation. However, we decided that it was conjecture rather than evidence and so removed it. Perhaps though we went too far. We have now extended the limitations paragraph (where this was originally mentioned) to highlight which recommendations would apply in different contexts. E.g. recommendation 3 is only applicable in countries with longitudinal healthcare data, but most of the others are general enough to be more widely applicable.
A second drawback is that the data are not publicly available, but the authors have done some effort to describe the necessary procedures. I think it is an open question whether other researchers from countries other than the UK will be able to get access to the data since only accredited researchers were allowed to have access.
The data used in this study was pseudonymised individual health records. These cannot be made publicly available under UK law. However any researcher wishing to access them can follow the application process – full details of which can be found in the Data Availability section.
